# The Effect of Psycho-Educational Interventions on the Quality of Life of the Family Caregivers of the Patients with Spinal Cord Injury: A Randomized Controlled Trial

**Published:** 2014-01

**Authors:** Zahra Molazem, Tayebeh Falahati, Iran Jahanbin, Peyman Jafari, Soraya Ghadakpour

**Affiliations:** 1 Community Based Psychiatric Care Research Center, Shiraz University of Medical Sciences, Shiraz, Iran;; 2 Student Research Committee, Shiraz University of Medical Sciences, Shiraz, Iran;; 3 Department of Biostatistics, Shiraz University of Medical Sciences, Shiraz, Iran;; 4 Department of Nursing, School of Nursing and Midwifery, Shiraz University of Medical Sciences, Shiraz, Iran

**Keywords:** Family Caregivers, Quality of Life, Psycho-Educational Intervention, Spinal Cord Injury

## Abstract

**Background:** Family caregivers usually report the reduction of their life quality due to one of the family member’s spinal cord injury. Thus, the present study aimed to investigate the effectiveness of psycho-educational interventions on the life quality of the family caregivers of the patients with spinal cord injury.

**Methods:** The present randomized controlled trial was conducted on 72 family caregivers who had the primary responsibility of taking care of the patients with spinal cord injury. The participants were randomly divided into intervention (n=36) and control groups (n=36). The intervention group was involved in 90-minute educational sessions held once a week for four weeks. Both groups completed SF-36 questionnaire before and 2 and 6 weeks after the intervention. Then, the data were analyzed through independent t-test, Chi-square, and repeated measures ANOVA.

**Results: **All the caregivers had low quality of life and the lowest mean score was related to mental health in both groups. After the intervention, various dimensions of life quality had improved in the intervention group’s caregivers compared to the control group (P<0.05).

**Conclusion: **The study results revealed the positive effect of psycho-educational interventions on the life quality of the caregivers of the patients with spinal cord injury. According to the results, the authorities have to pay special attention to the problems of this group and educational interventions have to be continuously followed.

**Trial Registration Number: **IRCT2013070811388N2

## Introduction

Spinal Cord Injury (SCI) is a complex phenomenon leading to bio-psychosocial changes which affect the patients’ as well as their caregivers’ health and life quality.^1^ No report of the accurate number of the SCI patients is available in Iran. However, 2.2 individuals in 10000 populations were affected in Tehran between 2003 and 2008.^2^ Besides, 29.5 people per million develop SCI worldwide every year.^3^ The problems related to SCI affect both the patients and their families; great changes occur in lives and responsibilities of the family members who take care of the patients suffering from SCI.^4^ In general, SCI causes physical,^5^ psychological,^6^ emotional,^7^ and economic^8^ problems for both the patients and their families. Although the disorder affects all the family members, the primary caregiver is responsible for providing physical, emotional, and financial care for the patients.^9^ Overall, caregivers play a critical role in improvement of the SCI patients.^4^

After the incidence of SCI, great changes occur in the family’s roles and dynamic status. In addition, the caregivers of such patients experience a lot of changes in their lifestyle and are considerably exposed to stress.^10^^,^^11^ These caregivers usually report the reduction of their life quality due to SCI.^12^ The major causes of stress among the caregivers of the patients suffering from SCI include the problems related to the consequences of paralysis, such as sexual dysfunction, limitations related to using wheelchair, change in the patients’ personality, and urinary system disorders.^13^ Moreover, researchers have reported a high level of physical as well as emotional stress, burnout, fatigue, anger, and depression among the caregivers of the SCI patients.^10^^,^^14^ Depressed mood, anxiety, tension, and fatigue are also among the factors which influence the individuals’ quality of life.^15^ In addition, chronic stress may lead to physical and mental problems which eventually affect the quality of care.^9^ In a large British longitudinal study, quality of life was considered as changes in life style and various impacts of stroke, including emotional distress, family relationships, and social involvement.^16^ In several studies, the extent of the physical disability of the stroke survivor was reported to be associated with the caregivers’ diminished quality of life or life satisfaction.^17^ Thus, paying special attention to the caregivers may improve their own, the patients’, and the whole families’ quality of life.^18^ Of course, the life quality of the family members who take care of the patients is far more important than that of the patients themselves.^19^^,^^20^ Therefore, the caregivers should be prepared to take care of the patients suffering from SCI through educational strategies because taking care of a dependent adult is quite boring and can put the caregiver’s health at a high risk.^1^ On the other hand, performing this stressful task without education or experience might decrease the quality of life and, at the same time, lead to communication problems and increase of stress among the family caregivers of the SCI patients.^12^^,^^21^ Thus, nurses have ethical and legal responsibility to prepare the SCI patients and their caregivers by executing educational programs.^1^

Up to now, no studies have been conducted on the life quality of the family caregivers of the patients suffering from SCI in Iran. Considering the lack of supportive systems for improving the family caregivers’ quality of life and reducing its related problems and taking the family caregivers’ important role in taking care of such patients into account, interventional studies have to be conducted in order to improve the life quality of the family caregivers of these patients. 

The present study aims to evaluate the effectiveness of psycho-educational interventions in the life quality of the family caregivers of the SCI patients.  

## Materials and Methods

**Figure 1:**
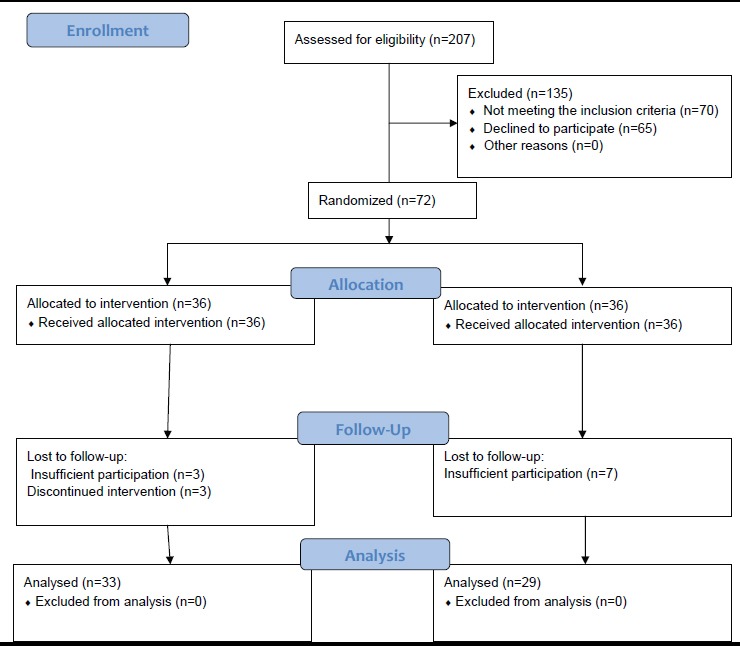
CONSORT Flow Diagram

The present randomized controlled trial was conducted on 72 family caregivers of SCI patients (paraplegic or tetraplegic) who had referred to the welfare organization of Shiraz in 2012 and met the inclusion criteria of the study. The design and protocol of the study have been shown in Figure 1. This study was conducted after obtaining license from the welfare organization of Shiraz, approval of the study by the Ethics Committee of Shiraz University of Medical Sciences, explaining the study methods and objectives to the caregivers, and obtaining written informed consents for taking part in the study.

All the participants completed the SF-36 questionnaire before and 2 and 6 weeks after the intervention. The data were collected from May until August 2012 and the individuals who had been the primary caregiver of a patient for one year and were willing to take part in the study were recruited into the research. Having access to telephone was also one of the inclusion criteria of the study. On the other hand, the exclusion criteria of the study were having a history of hospitalization, suffering from known mental disorders, and being under treatment by psychiatric medications.

The study participants were divided into two groups each containing 36 subjects through permuted-block randomization.^22^ The researcher divided the intervention group into three 12-subject groups and involved each in four 1.5 hour educational sessions which were held once a week for four consecutive weeks. The educational program was directed by one of the researchers who was the professor of psychiatric nursing in Shiraz University of Medical Sciences, Shiraz, Iran.

Also, at the end of the study, a booklet of the educational contents was given to the control group. However, 10 participants were excluded from the study (3 in the intervention group and 7 in the control group) because of insufficient participation or being on a trip. After all, the study was performed on 62 subjects, 33 in the intervention and 29 in the control group. 

The data were collected using SF-36 questionnaire before and 2 and 6 weeks after the intervention. SF-36 is a short 36-item questionnaire which evaluates 8 various dimensions of health; i.e., general health (5 items), physical function (10 items), limitation in role performance due to physical reasons (4 items), limitation in role performance due to emotional reasons (3 items), bodily pain (2 items), social function (2 items), vitality (4 items), and mental health (5 items). The minimum and maximum scores of this questionnaire are 0 and 100, respectively. Raw scores for each scale were transformed with an algorithm to a 0-100 scale. Different studies have reported the internal consistency of this questionnaire to range from 0.62 to 0.96. In addition, test-retest coefficients for the questionnaire range from 0.43 to 0.90.^23^ Its psychometric properties have also been evaluated in Iran revealing Cronbach’s α>%70.^24^ A researcher-made questionnaire including the demographic information was also completed by both study groups before the intervention.

All the statistical analyses were performed using the SPSS statistical software (v.15). The data were analyzed through t-test, Chi-square, and repeated measures ANOVA. Besides, P<0.05 was considered as statistically significant. 

At first, the participants of each study group got familiar with each other and the study objectives and took part in group discussions about the experience of living with an SCI patient. During these group discussions, the researcher encouraged the participants to freely exchange their thoughts and feelings so that they could actively investigate and understand their problems and feelings and more effectively deal with their problems by changing their attitudes and values.

Moreover, further sessions were arranged to train the participants regarding the strategies of coping with stress and depression, relaxation techniques, crisis confrontation strategies, principles of correct relationship within the family, and strategies for providing the SCI patients with correct physical care, preventing backache, and accurately transferring the patients from the bed to the wheelchair and vice-versa.

Psycho-educational intervention is an approach that provides information for the clients to be aware of the nature of their disease as well as the available treatment methods. The training skills that an individual can employ in life and society for one’s support are also considered as a part of such interventions.^25^ In this research, educational and psychological interventions were conducted through interactions among the group members. These interactions aimed at changing the participants’ attitudes and values so that they could deal with their problems more effectively.^26^

In this study, all the educational needs of the patients were evaluated and the content of the training program was developed according to other studies.^4^^,^^18^ The educational programs were presented through lecture, question and answer, and discussion using educational aids. The researcher’s phone number was also given to the caregivers for answering their probable questions.

## Results

Demographic characteristics of the study participants are presented in table 1. The results of t-test and Chi-square test revealed no significant relationship between the demographic variables and the caregivers’ quality of life dimensions in the two groups before the intervention (P>0.05).

**Table1:** Demographic characteristics of the study participants (N=62)

**Characters**	**Case ** **N(%)**	**Control** **N(**%)	**P value**
Sex			0.632
Male	2 (6.1)	1 (3.4)	
Female	31 (93.9)	28 (96.9)	
Marital status			1.00
Married	29 (87.9)	26 (89.7)	
Single	2 (6.1)	1 (3.4)	
Other	2 (6.1)	2 (6.9)	
Education level			0.646
Below high school	11 (33.3)	9 (31)	
High school	13 (39.4)	9 (31)	
Illiterate	9 (27.3)	11 (37.9)	
Occupation			0.460
Retired	1 (3)	0 (0)	
Retailer	0 (0)	2 (10.3)	
Housemaid	31 (94)	26 (89.7)	
Other	1 (3)	1 (3.4)	
Type of relationship			0.713
Parent	17 (51.5)	15 (51.7)	
Wife	12 (36.4)	12 (41.4)	
Sister	2 (6)	2 (6.9)	
Other	2 (6.1)	0 (0)	
Type of disability			0.739
Paraplegic	25 (75.8)	23 (79.3)	
Tetraplegic	8 (24.2)	6 (20.7)	
Cause of damage			0.646
Trauma	22 (66.7)	21 (72.4)	
Congenital	9 (27.3)	5 (17.2)	
Disease	2 (6.1)	3 (10.3)	
Age mean±SD	44.12±12.31	44.82±12.29	0.822
Length of times as a caregiver (years) mean±SD	9.39±6.68	9.65±6.74	0.879

SCI=Spinal Cord Injury; SD=Standard deviation; P<0.05 was considered statistically significant

The primary scores of various dimensions of the caregivers’ quality of life are presented in table 2. All the study participants had a low quality of life and the lowest score was related to mental health in both groups. After the intervention, various dimensions of the intervention group caregivers’ life quality had improved (P<0.05), while no significant difference was observed in the control group. Besides, a statistically significant difference was found between the two groups regarding all the dimensions of life quality (P<0.05) (table 3).

**Table 2:** Comparison of the mean scores of various dimensions of life quality in the two groups before the intervention by t-test (N=62).

**Dimensions**	**Case** **mean±SD**	**Control** **mean±SD**	**P value**
Physical function	40.83±8.97	37.88±9.61	0.216
Role physical	36.75±9.73	37.45±10.13	0.782
Bodily pain	37.57±9.65	35.86±11.68	0.529
General health	35.54±11.86	36.20±10.18	0.815
Vitality	43.18±10.78	44.34+11.02	0.677
Social function	34.19±11.24	38.22±12.74	0.191
Role emotional	33.31±11.94	38.86±10.03	0.054
Mental health	33.18±13.82	35.43±12.73	0.510

P values lower than 0.05 were considered statistically significant

**Table 3:** Comparing the dimensions of quality of life in the two groups during the study period by repeated measures ANOVA (N=62)

**Dimensions**	**Baseline**		**2 weeks**		**6 weeks**		**P value**
	**mean±SD**		**mean±SD**		**mean±SD**		**Time- group**
	**Intervention**	**Control**	**Intervention**	**Control**	**Intervention**	**Control**	
Physical function	40.83±8.97	37.88±9.61	46.36±7.33	41.37±7.05	47.56±6.76	37.86(8.53	0.003*
Role physical	36.75±9.73	37.88±9.61	47.86±6.29	38.37±8.11	48.42±8.04	38.21±9.92	0.001*
Bodily pain	37.57±9.65	35.86±11.68	42.93±9.58	38.11±8.96	47.11±9.41	37.85±9.09	0.019*
General health	35.54±11.86	36.20±10.18	44.64±10.49	37.45±9.22	46.41±8.76	36.87±10.19	0.001*
Vitality	43.18±10.78	44.34±11.02	51.06±9.16	43.40±9.25	51.51±9.29	42.51±10.85	0.001*
Social function	34.19±11.24	38.22±12.74	43.12±10.83	35.55±11.29	44.93±9.58	37.46±11.88	0.001*
Role emotional	33.31±11.94	38.86±10.03	46.70±8.68	38.58±9.66	46.45±9.17	35.67±10.02	0.001*
Mental health	33.18±13.82	35.43±12.73	44.31±11.56	32.73±10.67	47.45±10.53	33.68±13	0.001*

SCI=Spinal Cord Injury; SD=Standard deviation; *P values lower than 0.05 were considered as statistically significant

## Discussion

This study examined the short-term impact of psycho-educational interventions on the life quality of the family caregivers of the patients with SCI.

The study results showed that most of the participants were homemaker. The studies conducted in other communities have also shown that mostly girls and women take care of the SCI patients.^12^^,^^18^

According to the study results, the caregivers’ quality of life was low in all the dimensions. These results are in agreement with other studies.^12^^,^^27^ This confirms the necessity to pay attention to the problems the caregivers face in taking care of the SCI patients in daily life. Therefore, supportive systems have to be planned in order to investigate and follow the problems of this vulnerable group of the society.

The findings of the present study showed that the educational intervention was effective in all the life quality dimensions of the intervention group and improved their quality of life. However, no significant difference was observed in the control group (table 3). In the same line, the results of a study by Bell et al. showed that educational interventions accompanied by consultation had decreased the depression level of the caregivers with dementia and improved their quality of life.^28^ The educational and supportive program^29^ and the psycho-social intervention focusing on increase of knowledge and confrontation with problems and difficulties were also effective in improving the life quality of the caregivers of the patients suffering from dementia.^30^

After the intervention, the mean scores of bodily pain, general health, and mental health had increased in the intervention group caregivers. In general, therapeutic interventions, such as education, support, and psychotherapy can provide the ground for improvement of both physical and mental health.^31^ The results of another study showed that teaching problem solving could improve the life quality of the SCI patients’ family caregivers. They concluded that this psychological intervention increased the caregivers’ social function and had sedative effects on their physical function, as well.^32^ Moreover, increasing the support for SCI patients decreased their social isolation and encouraged them to control and improve their health.^18^ This was consistent with the results of the current study since the caregivers’ social and physical function was improved through applying the psycho-educational intervention. Thus, improvement of the caregivers’ vitality, bodily pain, general health, and mental health in this study can have resulted from the effect of the psycho-educational intervention on increasing the knowledge, paying attention to health, and decreasing the patients’ social isolation.

Nevertheless, the findings of this study were in contrast to those of another study indicating that the psychological education was effective in the caregivers’ quality of life in the group where both the patients and their caregivers were present. In the group where only the caregivers were present, the life quality had improved compared to the control group; however, the difference was not statistically significant.^18^ The difference between the results of the two studies might be due to the difference in the questionnaires, the caregivers’ cultural differences, and differences in the interventions’ contents and how they were performed. In this study, the caregivers were trained through face-to-face as well as group education, discussion, and lecture. In the study by Schulz et al., on the other hand, training was provided through the website.

One of the limitations of the present study was sampling from one center which is, of course, the main center in Shiraz; consequently, the results cannot be generalized to all the family caregivers. Another limitation of the study was its small sample size, which was due to the family caregivers’ high workload and lack of their cooperation. Thus, the researchers recommend more studies with larger sample sizes to be conducted on the issue in a longer period of time.

Overall, the findings of this study were encouraging and suggested that interventions, such as the family series workshop, had the potential to improve the health outcomes for the caregivers of the patients with SCI.

## Conclusion

The findings of the present study revealed the effectiveness of the psycho-educational intervention in improving the life quality of the caregivers of the SCI patients. Caregivers can benefit from the interventions that help them manage the mental and physical limitations. Thus, authorities and planners have to focus on the problems of this group of the society. Researchers should also make attempts to improve the life quality of the SCI patients’ family caregivers by performing educational, psychological, behavioral, and supportive interventions.
